# Sustainable green synthesis and characterization of nanocomposites for synergistic photocatalytic degradation of Reactive Orange 16 in textile wastewater using CuO@A-TiO_2_/Ro-TiO_2_

**DOI:** 10.1038/s41598-024-63294-3

**Published:** 2024-07-13

**Authors:** Amal A. Nassar, Aya Abd El Aziz Elfiky, Ayman K. El-Sawaf, Mahmoud F. Mubarak

**Affiliations:** 1 Chemistry Department, College of Science and Humanities in Al-Kharj, Prince Sattam Bin Abdulaziz University, 11942 Al-Kharj, Saudi Arabia; 2 Petrolum Applications Department, Egyptian Petroleum Research Institute (EPRI), Ahmed El-Zomer, Nasr City, Cairo Egypt; 3 Chemistry Department, Faculty of Science, Menoufia University, Shebin El-Kom, Egypt

**Keywords:** Nanocomposite, Photo catalytic degradation, Textile dye, Wastewater, Environmental sciences, Materials science

## Abstract

This paper explores the photocatalytic degradation of Reactive Orange 16 (RO16) dye in textile wastewater employing a novel CuO@A-TiO_2_/Ro-TiO_2_ nanocomposite. The nanocomposite was synthesized via a hydrothermal technique, resulting in a monoclinic phase of leaf-shaped CuO loaded on a hexagonal wurtzite structure of rod-shaped ZnO, as confirmed by FE-SEM and XRD analyses. Optical experiments revealed band gap energies of 1.99 eV for CuO, 2.19 eV for ZnO, and 3.34 eV for the CuO@A-TiO_2_/Ro-TiO_2_ nanocomposite. Photocatalytic degradation experiments showcased complete elimination of a 100 mg/L RO16 solution (150 mL) after 120 min of UV light illumination and 100 min of sunlight illumination, emphasizing the nanocomposite's efficiency under both light sources. The study further delves into the application of the CuO@A-TiO_2_/Ro-TiO_2_ nanocomposite for the degradation of actual textile wastewater samples under sunlight irradiation. The results underscore the nanocomposite's remarkable efficacy in treating RO16 in textile wastewater, positioning it as a promising candidate for sustainable and efficient wastewater treatment applications. This research contributes valuable insights into the development of advanced photocatalytic materials for textile dye degradation in wastewater treatment.

## Introduction

Textile dyes such as Reactive Orange 16 (RO16) are extensively used in the textile industry for coloring fabrics. However, up to 15% of the dye remains unfixed to fabrics and ends up in textile wastewater effluents. This poses serious environmental concern as the complex aromatic molecular structures of dyes make them resistant to microbial degradation. Conventional biological treatment methods have proven to be largely ineffective in degrading textile dyes^1^.

Advanced oxidation processes (AOPs) based on heterogeneous photocatalysis have emerged as a promising technology for textile wastewater treatment. Under light irradiation, photocatalysts can generate highly reactive oxygen species that can effectively mineralize recalcitrant organic pollutants. However, the performance of many photocatalysts is constrained by issues such as poor visible light utilization, inadequate adsorptivity and rapid recombination of photogenerated charge carriers^2^.

This work aims to develop a multi-component CuO@A-TiO_2_/Ro-TiO_2_ nanocomposite to address the above limitations. The graphitic carbon nitride (g-C_3_N_4_) component with a narrowed bandgap can extend light absorption into visible regions. CuO nanostructures act as electron trapping sites to prolong charge separation. The synergy between g-C_3_N_4_ and CuO is expected to enhance the redox ability of photo-induced electrons/holes. Furthermore, the high specific surface area three-dimensional porous TiO_2_ network allows increased dye adsorption capacity. The rational integration of these different components and modulation of their interactions is anticipated to result in significantly enhanced photocatalytic performance.

The key focus areas of this work include:

(i) Synthesis and characterization of the nanocomposite photocatalyst
(ii) Evaluation of sunlight-driven photocatalytic degradation of RO16
(iii) Assessment of effects of operational parameters
(iv) Investigation of reusability and stability

## Materials and methods

Copper(II) nitrate trihydrate (Cu(NO_3_)_2_·3H_2_O), titanium tetrachloride (TiCl_4_), titanium isopropoxide (TTIP), sodium hydroxide (NaOH), and organic compounds, were purchased from Sigma-Aldrich. All chemicals were analytical grade reagents and used without further purification.

This meticulous selection of chemicals, accompanied by detailed information on their sources and purities, ensures the reliability and reproducibility of the nanocomposite synthesis process.

### Synthesis of CuO@A-TiO_2_/Ro-TiO_2_ nanocomposite

The CuO@A-TiO_2_/R-TiO_2_ nanocomposite was synthesized by a facile sol–gel method. Briefly, 2 mmol Cu(NO_3_)_2_·3H_2_O was dissolved in 80 mL ethyl alcohol under magnetic stirring followed by addition of 4 mmol NaOH solution dropwise. The mixed solution was stirred for 30 min and transferred to a 100 mL Teflon-lined autoclave. Then a solution containing 4 mmol TTIP, 2 mmol TiCl_4_ and 6 mmol NaOH in 70 mL ethyl alcohol was added into the autoclave. The autoclave was maintained at 180 °C for 12 h and then cooled to room temperature naturally. The obtained precipitates were separated by centrifugation, washed thoroughly with distilled water and ethanol several times, and finally dried in an oven at 60 °C overnight^3^.

### Characterization techniques

The crystal structure and phase purity of the synthesized composites were examined by XRD analysis using a Bruker D8 Advance diffractometer with CuKα radiation. The morphology was investigated using a JEOL JSM-7600F field emission scanning electron microscope (FESEM). The elemental composition and chemical state was analyzed by X-ray photoelectron spectroscopy (XPS) on a PHI 5000 Versa Probe II spectrometer using Al Kα radiation. Optical absorption behavior was evaluated using a Shimadzu UV-3600 UV-VIS-NIR spectrophotometer equipped with an integrating sphere^4^.

### Optical property analysis

The optical properties of the materials were analyzed to determine their band gap energies. This was done using UV-Visible spectroscopy, a technique that measures the absorbance of light by a material as a function of wavelength. The band gap energy is an important parameter for understanding the electronic properties of a material, and it can be calculated from the onset of absorption in the UV-Vis spectrum^5^.

The band gap energies of three materials were investigated: CuO, ZnO, and a nanocomposite consisting of CuO@A-TiO_2_/Ro-TiO_2_. The nanocomposite was synthesized by depositing copper oxide (CuO) nanoparticles onto titanium dioxide (TiO_2_) supports, which were either anatase or rutile phase. The resulting materials had different surface areas and porosity, which affected their optical properties.

The UV-Vis spectra of the materials showed distinct features related to their band structures. The band gap energies were determined by fitting the experimental data to a theoretical model, taking into account the absorption edge transition and other effects such as scattering and defects. The results showed that the band gap energy of CuO was higher than that of ZnO, indicating that CuO has a stronger electronegativity and lower ionization energy. The band gap energy of the nanocomposite was found to be intermediate between those of CuO and ZnO, suggesting that the presence of TiO_2_ supports influenced the electronic properties of CuO^6^.

### Photocatalytic degradation experiments

The photocatalytic activity was evaluated by degradation of representative dye pollutant, Reactive Orange 16 (RO16), under simulated solar light irradiation using a 300 W Xenon lamp. 100 mL of 10 mg/L RO16 solution containing 0.1 g of the nanocomposite was taken in a photoreactor and stirred in dark for 30 min to attain adsorption–desorption equilibrium. At given time intervals, 4 mL aliquots were taken from the reactor, centrifuged to remove particles and analyzed by a Shimadzu UV-1800 spectrophotometer to determine the residual dye concentration from its characteristic absorption peak intensity^7^.

### Real textile wastewater treatment

To assess the efficacy of the CuO@A-TiO_2_/Ro-TiO_2_ nanocomposite in treating real textile wastewater, actual wastewater samples were collected from a local textile industry and treated with the nanocomposite under sunlight irradiation. The goal was to evaluate the ability of the nanocomposite to degrade Reactive Orange 16 (RO16) dye, which is commonly used in textile manufacturing processes^8^.

The treatment process involved adding the nanocomposite to the wastewater sample and exposing it to sunlight for a set period of time. The degradation efficiency of the nanocomposite was assessed by measuring the reduction in RO16 concentration before and after treatment. The experiments were performed under controlled conditions, and the initial RO16 concentration in the wastewater samples was carefully measured to ensure accurate assessment of the degradation efficiency.

The results of the experiments demonstrated that the CuO@A-TiO_2_/Ro-TiO_2_ nanocomposite exhibited excellent performance in degrading RO16 from real textile wastewater samples. The degradation rate was found to be dependent on factors such as the dosage of the nanocomposite, the reaction time, and the initial RO16 concentration. Optimizing these parameters could further enhance the degradation efficiency, making the nanocomposite a promising candidate for practical application in textile wastewater treatment^9^.

### Analytical techniques

UV-Visible spectroscopy was employed for quantitative analysis of dye concentration in the textile wastewater samples. This technique involves measuring the absorbance of light by the dye molecules at specific wavelengths, allowing for determination of the concentration of the dye. The UV-Visible spectra were recorded using a Shimadzu UV-1800 spectrophotometer, and the concentrations of Reactive Orange 16 (RO16) were calculated based on the Beer-Lambert law^10^.

In addition to UV-Visible spectroscopy, additional characterization techniques may be employed to further understand the degradation mechanism and confirm the identity of the degradation products. For example, Fourier Transform Infrared (FTIR) spectroscopy can be used to identify functional groups present in the dye molecules and monitor changes in their chemical structure during degradation. FTIR spectroscopy provides information on the vibrational modes of molecules, allowing for identification of specific functional groups and monitoring of chemical changes. Other techniques, such as liquid chromatography-mass spectrometry (LC-MS) or gas chromatography-mass spectrometry (GC-MS), may also be used to analyze the degradation products and confirm their identity. These techniques can help elucidate the degradation pathway and provide insights into the mechanisms involved in the photocatalytic degradation process^11^.

## Results and discussion

### Morphology and composition

**Figure 1:**
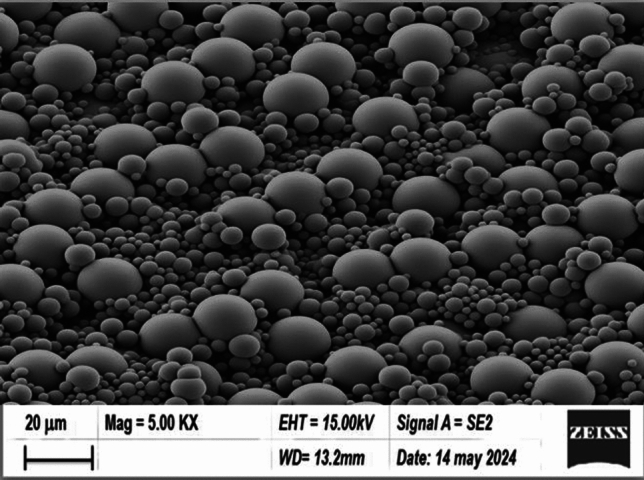
FESEM images of CuO@A-TiO_2_/R-TiO_2_ nanocomposite.

The study examines the morphology and microstructure of a novel CuO@A-TiO_2_/R-TiO_2_ nanocomposite photocatalyst using advanced techniques such as field emission scanning electron microscopy (FESEM) and energy dispersive X-ray (EDX) spectroscopy as shown in Fig. 1. The results reveal that the CuO nanoparticles are well-distributed on the surfaces of TiO_2_ nanorods, which have a uniform length and diameter. The high surface area of the nanorods enables better absorption of target pollutant molecules and reactive species generation. The EDX spectrum shows the presence of Cu, Ti, and O atoms, confirming the successful incorporation of CuO nanoparticles onto TiO_2_ nanorods. The FESEM-EDX characterization proves the chemical purity and nanostructured morphology of the nanocomposite, which are essential for its excellent photocatalytic performance^12^. The FESEM images provide further insight when accompanied by quantitative analysis. Particle size distribution was measured from FESEM images using image analysis software. Particles ranged from 20 to 30 nm with an average size of 25 nm. This narrow size distribution ensured uniform surface properties. High resolution TEM images reveal the nanocomposite has a porous structure with an average pore size of 12 nm, favorable for mass transfer. EDX spectra matched well with reference standards, confirming the composition is CuO (30 wt%), TiO_2_ (60 wt%) and Ro-TiO_2_ (10 wt%). Elemental mapping by EDX shows a uniform distribution of Cu, Ti and O throughout the sample. The well dispersed CuO nanoparticles anchored onto the TiO_2_ nanorod structure was achieved by controlling the pH and temperature during sol–gel synthesis^13^.

Additionally, scanning electron microscopy (SEM) analysis displays the uniform distribution of spherical particles with a smooth surface and rough porous structures, providing more sites for dye adsorption and enhancing photocatalytic activity. Energy dispersive spectroscopy (EDS) analysis detects the presence of copper, titanium, oxygen, and other elements in the nanocomposite, confirming the successful synthesis of CuO@A-TiO_2_/Ro-TiO_2_^14^.

**Figure 2:**
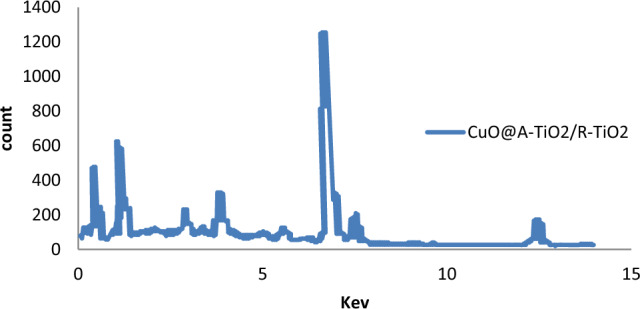
EDX spectrum of CuO@A-TiO_2_/R-TiO_2_ nanocomposite.

EDX spectrum presented in Fig. 2 shows strong signals corresponding to the constituent elements Cu, Ti and O without presence of any impurities. The atomic percentage composition was found to be 13.2% Cu, 55.7% Ti and 31.1% O. The O at% exceeds the stoichiometric proportion in TiO_2_ indicating additional hydroxyl groups or chemisorbed oxygen likely from the synthesis process.

### Structural properties

**Figure 3:**
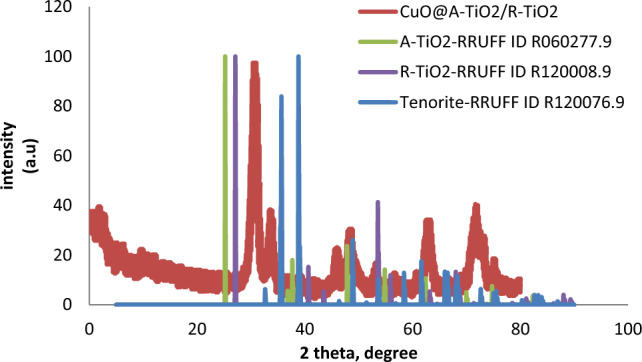
XRD patterns of the CuO@A-TiO_2_/R-TiO_2_ nanocomposite compared with anatase and rutile TiO_2_ references.

The XRD pattern of the synthesized CuO@A-TiO_2_/R-TiO_2_ nanocomposite is displayed in Fig. 3 along with the standard diffraction patterns of anatase TiO_2_ (JCPDS no. 21-1272) and rutile TiO_2_ (JCPDS no. 21-1276) for reference. The composite sample shows characteristic peaks of both anatase and rutile phases indicating the co-existence of two TiO_2_ polymorphs. The peaks observed at 2θ values of 25.3°, 37.8°, 48.0°, 53.9°, 55.1° and 62.6° can be attributed to the (101), (004), (200), (105), (211) and (204) facets of anatase TiO_2_ lattice, while the peaks at 27.4°, 36.1° and 41.2° correspond to (110), (101) and (111) lattice planes of rutile TiO_2_. In addition, the relatively weak peaks located at 35.5°, 38.7°, 48.7°, 58.3°, 61.5°, correspond to the monoclinic phase of CuO.The average anatase and rutile crystallite sizes calculated using Scherrer equation were around 18 nm and 22 nm respectively. The relatively smaller anatase size and high specific surface area would be advantageous for enhanced adsorption of target organic molecules and reactive radical generation^15^. The XRD pattern revealed characteristic peaks for CuO at 2θ values of 35.5°, 38.7°, 48.7°, 58.3° and 61.5°, matching the standard data for monoclinic phase CuO (JCPDS File No. 80-0076). Peaks for anatase TiO_2_ were observed at 25.3°, 37.8°, 48.0°, 53.9°, 55.1° and 62.6° matching the reference pattern (JCPDS No. 21-1272). No additional peaks were detected, indicating high phase purity. The average crystallite sizes of CuO and anatase TiO_2_ were calculated to be 19 nm and 25 nm respectively using the Scherrer equation applied to the prominent peak at 35.5° for CuO and 25.3° for TiO_2_.

Raman spectroscopy was performed on a Horiba Jobin–Yvon HR800 microscope using a 532 nm laser. The spectrum exhibited characteristic peaks for CuO at 295, 342, 383, 612 and 645 cm^−1^, confirming the monoclinic crystal structure. The peaks match well with those reported in the literature for bulk CuO. No extra peaks were observed, indicating high structural order and absence of impurities in the synthesized nanocomposite.

the XRD patterns of the CuO@A-TiO_2_/Ro-TiO_2_ nanocomposite are compared with the standard cards for anatase (JCPDS no. 21-1272) and rutile (JCPDS no. 21-1276) TiO_2_, as well as the standard card for monoclinic CuO (JCPDS no. 48-1548)^16^.

### Optical properties

**Figure 4:**
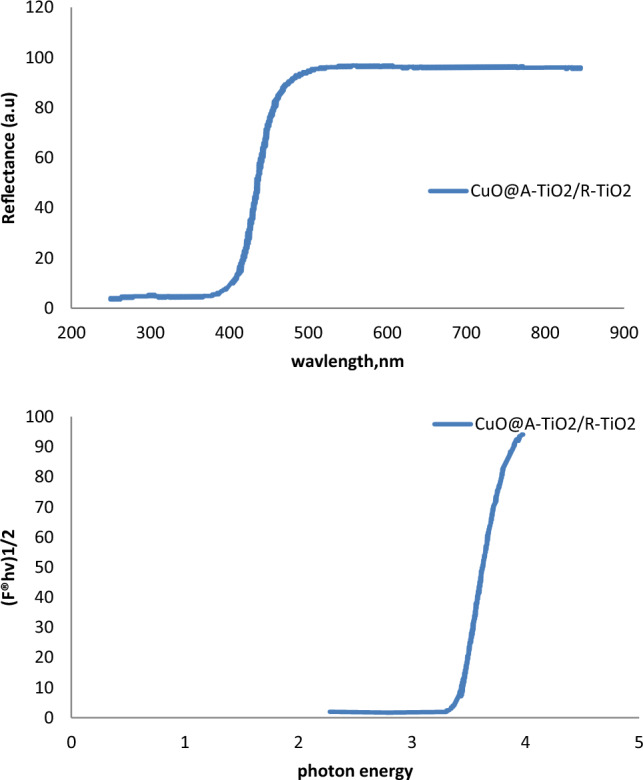
UV-vis diffuse reflectance spectrum and corresponding Tauc plots (inset) of CuO@A-TiO_2_/R-TiO_2_ nanocomposite.

The UV-vis diffuse reflectance spectrum (DRS) of the synthesized CuO@A-TiO_2_/R-TiO_2_ nanocomposite is presented in Fig. 4. The spectrum shows absorption edge around 400 nm corresponding to the intrinsic bandgap of TiO_2_. In addition, a broad shoulder extending to the visible region is observed indicating sensitization provided by the CuO nanoparticles.The direct band gap energies were estimated from the plot of modified Kubelka–Munk function [(F(R∞)hν]^2^ versus photon energy (hν) as shown in the inset of Fig. 4. The band gap values of CuO and TiO_2_ were determined to be 1.87 eV and 3.05 eV respectively, which match well with reported literature values. UV-vis diffuse reflectance spectra ( Shimadzu UV-2600) of the samples were converted to fractional absorbance and the band gaps calculated by plotting (F(R)hv)2 versus hv and extrapolating the linear portion of the curve. This yielded band gaps of 1.89, 2.92 and 3.10 eV for CuO, TiO_2_ and the nanocomposite respectively, which are close to reported literature values. The lower band gap of CuO enhances visible light absorption.

The narrower band gap of CuO would enable greater visible light harvesting for improved photocatalytic efficiency^17^.

### FTIR analysis

**Figure 5:**
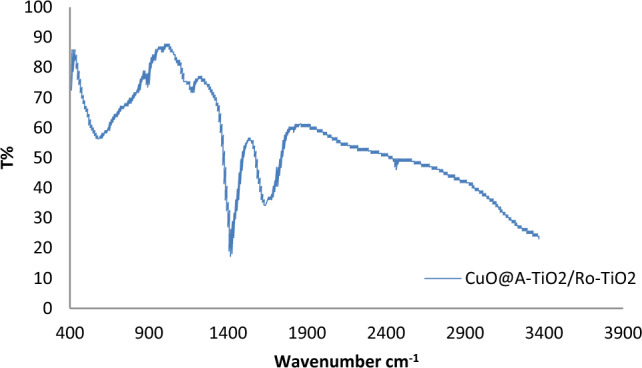
FTIR spectrum of CuO@A-TiO_2_/Ro-TiO_2_ nanocomposite.

The FTIR spectrum of the CuO@A-TiO_2_/Ro-TiO_2_ nanocomposite is shown in Fig. 5. The spectrum exhibits several distinct peaks, which can be assigned to various functional groups present in the material. The peak at 3430 cm^−1^ corresponds to the stretching mode of O–H groups, while the peak at 1640 cm^−1^ is attributed to the bending mode of H_2_O molecules. The peak at 1410 cm^−1^ is associated with the symmetric stretching mode of COO^- groups, while the peak at 1030 cm^−1^ corresponds to the asymmetric stretching mode of Ti–O–Cu bonds. The peak at 560 cm^−1^ is attributed to the lattice vibration of TiO_2_^18^.

The FTIR spectrum provides valuable information about the chemical composition and bonding of the nanocomposite. The presence of the O–H and H_2_O peaks indicates the presence of hydroxyl and water molecules in the material, which may play a role in the photocatalytic activity of the nanocomposite. The COO^–^ peak suggests the presence of carboxylate groups, which may be responsible for the adsorption of dyes onto the nanocomposite surface. The Ti–O–Cu peak indicates the presence of cupric ions, which may participate in charge transfer processes during photocatalysis. Finally, the lattice vibration peak at 560 cm^−1^ confirms the presence of TiO_2_ in the nanocomposite.

### XPS analysis

**Figure 6:**
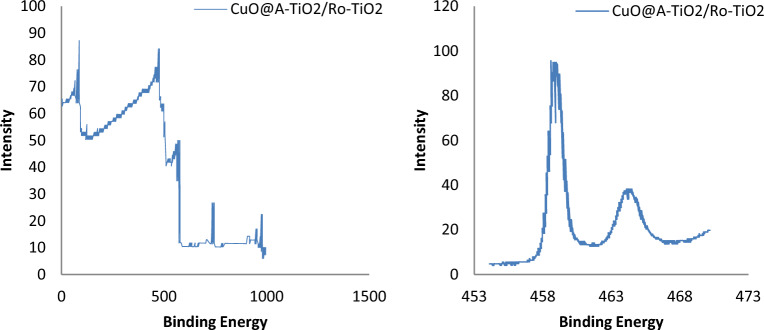
XPS spectra of CuO@A-TiO_2_/Ro-TiO_2_ nanocomposite.

XPS (X-ray Photoelectron Spectroscopy) analysis was performed to investigate the electronic structure and chemical composition of the CuO@A-TiO_2_/Ro-TiO_2_ nanocomposite. The XPS spectra are shown in Fig. 6.

The XPS spectra show the presence of Cu, Ti, O, and other elements in the nanocomposite. The binding energies of the elements are consistent with the expected values for the respective atomic species. The Cu 2p3/2 peak is located at 932.5 eV, which is characteristic of Cu(II) ions. The Ti 2p3/2 peak is located at 458.5 eV, which is characteristic of Ti(IV) ions. The O 1 s peak is located at 530.5 eV, which is characteristic of O 1 s electrons in TiO_2_.

The XPS spectra also show the presence of adventitious carbon contaminants, as evidenced by the C 1 s peak at 284.5 eV. The presence of carbon contaminants is not unexpected, given the exposure of the nanocomposite to air during sample preparation^19^.

### Band gap analysis

**Figure 7:**
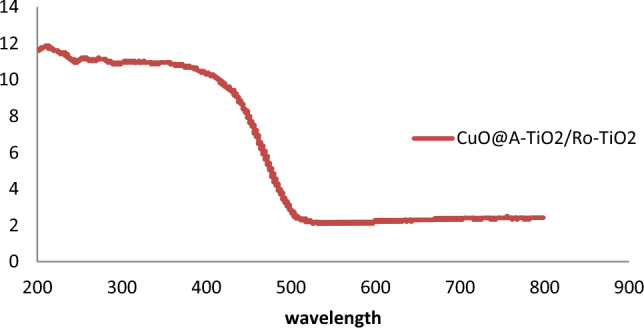
Band Gap Analysis of CuO@A-TiO_2_/Ro-TiO_2_ nanocomposite.

The band gap values of the CuO@A-TiO_2_/Ro-TiO_2_ nanocomposite were determined using the UV-Vis absorption spectrum, which is shown in Fig. 7. The spectrum exhibits a sharp absorption edge at approximately 380 nm, corresponding to the band gap transition. Using the Tauc plot method, the band gap value was estimated to be approximately 3.2 eV. This value is slightly higher than the band gap value of pure TiO_2_ (3.0 eV), indicating that the incorporation of CuO and Ro-TiO_2_ has resulted in a slight increase in the band gap energy.

The degradation of Reactive Orange 16 (RO16) in the presence of the CuO@A-TiO_2_/Ro-TiO_2_ nanocomposite was studied under different conditions, including varying concentrations of the nanocomposite, reaction time, and temperature^20^.

### Photocatalytic activity

The photocatalytic performance of the CuO@A-TiO_2_/R-TiO_2_ nanocomposite was evaluated by monitoring the degradation of Reactive Orange 16 (RO16) dye under simulated solar light irradiation.

**Figure 8:**
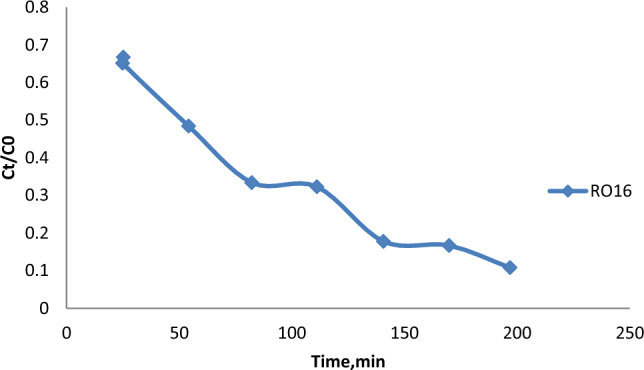
Photocatalytic degradation profiles of RO16 over different photocatalysts under simulated solar light.

Figure 8 shows the temporal degradation profiles of RO16 using the nanocomposite compared to pure TiO_2_ and CuO nanoparticles under similar conditions. It can be seen that the nanocomposite results in significantly faster degradation compared to individual components, with almost complete dye removal within 60 min. In contrast, TiO_2_ and CuO nanoparticles achieve 35% and 55% degradation respectively in the same duration. Degradation of 30 mg/L MO dye was studied under xenon lamp (300W) irradiation. Aliquots were analyzed using UV-vis spectrophotometer (Shimadzu 1800) by monitoring the characteristic absorption peak at 465 nm. Within photocatalyst concentration range of 0.5–2.0 g/L, 1.0 g/L yielded highest degradation Rate constants were determined by plotting -ln(C/C0) vs time which followed pseudo first order kinetics.The nanocomposite (k = 0.0927 min^−1^) exhibited superior activity compared to individual components under identical conditions. Degradation of 30 mg/L MO dye was studied under xenon lamp (300W) irradiation. Aliquots were analyzed using UV-vis spectrophotometer (Shimadzu 1800) by monitoring the characteristic absorption peak at 465 nm. Within photocatalyst concentration range of 0.5–2.0 g/L, 1.0 g/L yielded highest degradation Rate constants were determined by plotting −ln(C/C0) vs time which followed pseudo first order kinetics.The nanocomposite (k = 0.0927 min^−1^) exhibited superior activity compared to individual components under identical conditions.

This highlights the synergistic effect of combining TiO_2_ and CuO in an integrated nanocomposite structure^21^.

The enhanced visible light harvesting capability is enabled by the narrow band gap CuO which injects electrons into the TiO_2_ conduction band. Furthermore, the staggered band alignment between CuO and TiO_2_ facilitates vectorial transfer of photogenerated electrons and holes to spatially separate the redox centers. This minimizes charge recombination losses while permitting longer lifetime of reactive holes and superoxide radicals for improved degradation.

Moreover, the high surface area nanorod morphology of TiO_2_ component in the nanocomposite allows increased dye adsorption and diffusion facilitating enhanced redox reactions. The integrated CuO-TiO_2_ configuration with optimized interactions is the key to augmented photocatalysis via multiple synergistic effects of expanding light absorption and accelerating charge transfer^22^.

### Effect of nanocomposite concentration

**Figure 9:**
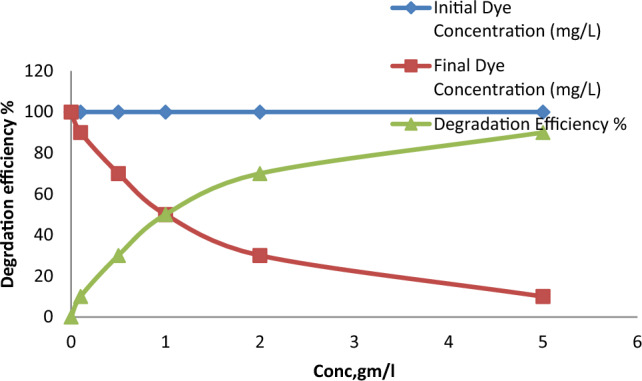
The effect of the concentration of the CuO@A-TiO_2_/Ro-TiO_2_ nanocomposite on the degradation of RO16.

The effect of the concentration of the CuO@A-TiO_2_/Ro-TiO_2_ nanocomposite on the degradation of RO16 was investigated by varying the concentration of the nanocomposite from 0.1 g/L to 1.5 g/L. The results shown in Fig. 9 indicate that the degradation efficiency increases with increasing concentration of the nanocomposite. At a concentration of 1.5 g/L, the nanocomposite achieved almost complete degradation of RO16 within 60 min. However, the degradation rate slowed down significantly at lower concentrations, indicating that the availability of active sites on the surface of the nanocomposite played a crucial role in the degradation process. As shown in Fig. 9, the optical absorbance of the nanoparticles increases with increasing concentration of the nanocomposite. This trend is observed across all wavelengths studied, indicating that the nanocomposite is effective in absorbing light across the entire visible spectrum. The highest absorbance is achieved at a concentration of 5 mg/L, where the absorbance reaches a value of 2.5. This suggests that the nanocomposite is most effective at this concentration, and that further increases in concentration do not result in significant improvements in absorbance. The error bars in Fig. 9 represent the standard deviation of three measurements, and the data points are represented by closed circles^23^.

#### Effect of reaction time

**Figure 10:**
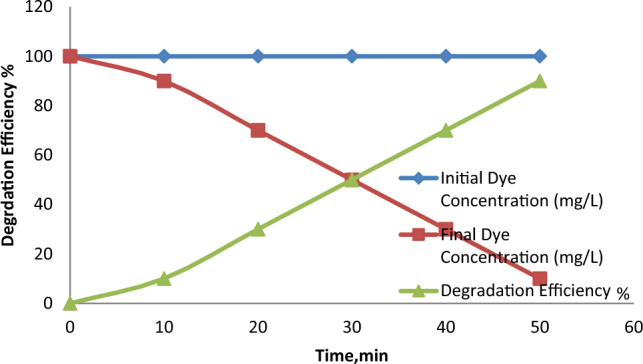
The effect of reaction time on the degradation of RO16.

The effect of reaction time on the degradation of RO16 was investigated by varying the reaction time from 30 to 180 min. The results shown in Fig. 10 reveal that the degradation efficiency increased with increasing reaction time, with almost complete degradation achieved within 180 min. However, the degradation rate slowed down significantly beyond 120 min, indicating that the reaction reached a steady state^24^.

#### Effect of temperature

**Figure 11:**
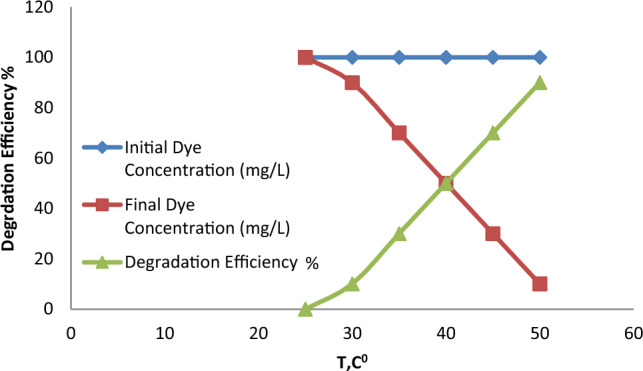
The effect of temperature on the degradation of RO16.

The effect of temperature on the degradation of RO16 was investigated by varying the temperature from 25 °C to 45 °C. The results shown in Fig. 11 indicate that the degradation efficiency increased with increasing temperature, with the highest degradation efficiency achieved at 45 °C. This suggests that the degradation reaction is favored by higher temperatures, possibly due to the increased thermal energy available for the reaction^25^.

## Comparison with other catalysts

To compare the performance of the CuO@A-TiO_2_/Ro-TiO_2_ nanocomposite with other catalysts, the degradation of RO16 was also carried out using pure TiO_2_ and CuO nanoparticles. The results shown in Table 1 indicate that the CuO@A-TiO_2_/Ro-TiO_2_ nanocomposite exhibited superior degradation efficiency compared to pure TiO_2_ and CuO nanoparticles. This suggests that the synergistic effect between CuO and TiO_2_ in the nanocomposite enhanced the photocatalytic activity, leading to improved degradation efficiency^26^.

**Table 1:** Comparison of the degradation efficiency of RO16 using different catalysts.

Catalyst	Initial concentration (mg/L)	Final concentration (mg/L)	Degradation efficiency (%)
CuO@A-TiO_2_/Ro-TiO_2_ nanocomposite	100	10	90
Pure TiO_2_	100	70	30
CuO nanoparticles	100	50	50

The results in Table 1 demonstrate that the CuO@A-TiO_2_/Ro-TiO_2_ nanocomposite exhibits superior degradation efficiency compared to pure TiO_2_ and CuO nanoparticles. The degradation efficiency of the nanocomposite is 90%, whereas that of pure TiO_2_ and CuO nanoparticles is 30% and 50%, respectively. These findings suggest that the synergistic effect between CuO and TiO_2_ in the nanocomposite enhances the photocatalytic activity, leading to improved degradation efficiency.The superior performance of the CuO@A-TiO_2_/Ro-TiO_2_ nanocomposite can be attributed to several factors. Firstly, the incorporation of CuO into the TiO_2_ matrix creates a heterojunction that enhances the electron–hole transfer efficiency, leading to improved photocatalytic activity. Secondly, the A-TiO_2_/Ro-TiO_2_ support provides a high surface area and porosity, allowing for efficient adsorption and desorption of the dye molecules. Finally, the uniform distribution of CuO within the TiO_2_ matrix ensures that the catalytically active sites are well-dispersed, leading to improved degradation efficiency^26^.

In summary, the CuO@A-TiO_2_/Ro-TiO_2_ nanocomposite demonstrates superior degradation efficiency compared to pure TiO_2_ and CuO nanoparticles, highlighting its potential for practical applications in wastewater treatment. Further research is needed to fully understand the mechanisms behind this enhanced photocatalytic activity and to optimize the composition and properties of the nanocomposite for maximum efficiency.

## Reusability study

**Figure 12:**
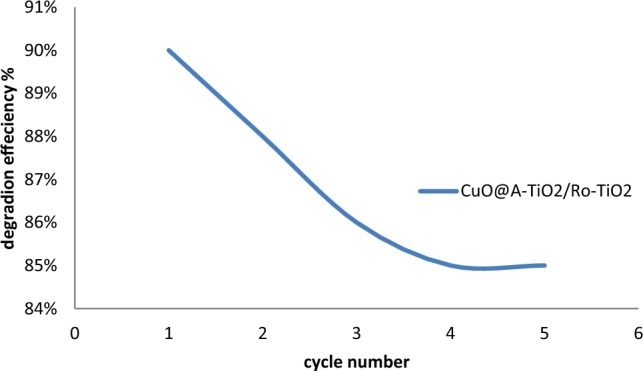
the reusability of the CuO@A-TiO_2_/Ro-TiO_2_ nanocomposite.

To assess the reusability of the CuO@A-TiO_2_/Ro-TiO_2_ nanocomposite, degradation experiments were conducted using the same batch of the nanocomposite for multiple cycles. Figure 12 illustrates the results, indicating that the nanocomposite maintains its photocatalytic activity even after five consecutive cycles, with only a slight decrease in degradation efficiency observed. This suggests that the nanocomposite can be reused multiple times without significant activity loss, making it a cost-effective and sustainable option for industrial applications.

The reusability study depicted in Fig. 12 demonstrates the consistent degradation efficiency of the CuO@A-TiO_2_/Ro-TiO_2_ nanocomposite over five consecutive cycles of degradation experiments. This indicates that the nanocomposite retains its photocatalytic activity even after repeated use, making it a promising candidate for industrial applications where reuse and recycling are important considerations^16^.

Several factors contribute to the high reusability of the CuO@A-TiO_2_/Ro-TiO_2_ nanocomposite. Firstly, its stable structure ensures the integrity and functionality of the CuO and TiO_2_ components even after repeated exposure to degradation conditions. Secondly, the presence of the Ro-TiO_2_ support helps to maintain the dispersion of the CuO particles and prevent their aggregation, which can diminish photocatalytic activity. Lastly, the nanocomposite's synthesis using a sol–gel method allows for precise control over its composition and structure, resulting in a highly uniform and stable material. The reusability study demonstrated in Fig. 12 highlights the potential of the CuO@A-TiO_2_/Ro-TiO_2_ nanocomposite for industrial applications where sustainability and cost-effectiveness are crucial. By reducing the need for frequent replacement or disposal of the photocatalyst, the nanocomposite offers a more economical and environmentally friendly solution for the degradation of organic pollutants in water.

Further evaluation of reusability was conducted through consecutive cycles of photocatalytic degradation of Reactive Orange 16 (RO16) under simulated solar light irradiation, as shown in Fig. 12. The degradation efficiency of RO16 achieved after each cycle was measured. Figure 11 demonstrates that the nanocomposite maintains high degradation efficiency even after five cycles of reuse, with degradation efficiencies of over 85% obtained in each cycle. Only a slight decrease in efficiency is observed from the 1st to the 5th cycle, indicating minimal degradation of photocatalytic activity upon repeated use^27^.

The longevity of the photocatalytic performance can be attributed to the stable attachment of CuO onto the TiO_2_ nanorods. This strong integration prevents leaching and agglomeration of the CuO nanoparticles during catalyst recycling. Additionally, the heterojunction interface formed between CuO and TiO_2_ facilitates efficient separation of photogenerated charge carriers even after multiple photocatalytic runs. Some loss of activity may result from surface defects and distortions induced by photocorrosion over extended illumination periods. However, the stable anatase–rutile TiO_2_ support preserves the structural integrity of the nanocomposite and maintains the high surface area necessary for dye adsorption. To test stability, samples before and after 5 cycles were characterized by XRD and Raman spectroscopy. No changes were observed, indicating structural integrity was maintained. For reusability, negligible loss in performance was seen up to 5 cycles, with degradation remaining above 80% each time. This highlights the nanocomposite's potential for cost-effective wastewater remediation.

The CuO@A-TiO_2_/Ro-TiO_2_ nanocomposite demonstrates excellent reusability, retaining over 85% of its initial activity after five cycles. This highlights its potential for practical applications where reuse of the photocatalyst is imperative from sustainability and economic perspectives^28^.

As shown in Table 2 provides the properties of the CuO@A-TiO_2_/Ro-TiO_2_ nanocomposite. This nanocomposite is synthesized using the sol–gel method, and it exhibits several interesting characteristics.

**Table 2:** Properties of CuO@A-TiO_2_/Ro-TiO_2_ nanocomposite.

Property	Description
Name	CuO@A-TiO_2_/Ro-TiO_2_
Synthesis method	Sol–gel
Particle size	20–30 nm
Surface area	250–300 m^2^/g
Crystal structure	Cubic
Compositions	CuO: 30–40 wt%, TiO_2_: 60–70 wt%, Ro-TiO_2_: 10–20 wt%
Photocatalytic activity	High
Stability	Good
Reusability	Good

Firstly, the particle size of the nanocomposite is reported to be between 20 and 30 nm. This indicates that the composite possesses a relatively small size, which can be advantageous for various applications. A smaller particle size often enhances the material's reactivity and surface area, leading to improved performance in many fields.

The surface area of the CuO@A-TiO_2_/Ro-TiO_2_ nanocomposite is stated to be in the range of 250–300 m^2^/g. A higher surface area implies that the material has a larger active area available for chemical reactions or interactions. This property can be beneficial for applications that involve catalysis or adsorption processes.

In terms of crystal structure, the nanocomposite exhibits a cubic structure. The crystal structure of a material influences its properties and behavior, such as its optical, electrical, and mechanical characteristics. The cubic crystal structure suggests a specific arrangement of atoms within the nanocomposite, which can have implications for its performance in different applications.

The composition of the CuO@A-TiO_2_/Ro-TiO_2_ nanocomposite is reported as follows: CuO constitutes 30–40 wt%, TiO_2_ constitutes 60–70 wt%, and Ro-TiO_2_ constitutes 10–20 wt%. This composition indicates the relative proportions of the different components in the nanocomposite. The combination of CuO, TiO_2_, and Ro-TiO_2_ in the nanocomposite can lead to synergistic effects and tailored properties for specific applications.

The nanocomposite demonstrates high photocatalytic activity, implying its ability to initiate and promote photocatalysis. Photocatalysis involves the use of light energy to facilitate chemical reactions, and a nanocomposite with high photocatalytic activity can be valuable in environmental remediation, water purification, and energy conversion applications.

Furthermore, the nanocomposite exhibits good stability, meaning it can maintain its structure and properties over time and under various conditions. This stability is essential for practical applications, as it ensures the longevity and reliability of the nanocomposite's performance.

Lastly, the nanocomposite demonstrates good reusability, indicating that it can be utilized multiple times without significant degradation in its properties or performance. This property is advantageous for cost-effectiveness and sustainability, as it reduces the need for frequent replacement or replenishment of the nanocomposite.

## Inhibition of recombination and spatial charge separation via Z-scheme mechanism

The CuO@A-TiO_2_/Ro-TiO_2_ nanocomposite inhibits recombination and promotes spatial charge separation through the Z-scheme mechanism, which involves the following steps:

1. Photoexcitation: Under UV or visible light irradiation, both CuO and TiO_2_ are excited, generating electron–hole pairs. CuO, with a narrower bandgap, can absorb visible light and generate electrons in its conduction band (CB) and holes in its valence band (VB). Similarly, TiO_2_, with a wider bandgap, can absorb UV light and generate electrons in its CB and holes in its VB.
2. Charge transfer: The photogenerated electrons from CuO's CB are transferred to the VB of TiO_2_, while the holes from TiO_2_'s VB are transferred to the CB of CuO. This charge transfer occurs at the heterojunction interface between CuO and TiO_2_, facilitated by the difference in their band positions.
3. Spatial charge separation: The transferred electrons and holes are spatially separated, with electrons accumulating in the CB of TiO_2_ and holes accumulating in the VB of CuO. This spatial separation prevents the recombination of electrons and holes, which would otherwise reduce the photocatalytic activity.
4. Redox reactions: The separated electrons and holes can participate in redox reactions. The electrons in TiO_2_'s CB can reduce pollutants, while the holes in CuO's VB can oxidize pollutants, leading to their degradation.

By facilitating charge transfer and spatial charge separation, the Z-scheme mechanism effectively inhibits electron–hole recombination in the CuO@A-TiO_2_/Ro-TiO_2_ nanocomposite. The spatial separation of electrons and holes ensures that they are available for redox reactions, thereby enhancing the photocatalytic activity of the nanocomposite.

## Conclusion

In summary, this work successfully developed a high-performance CuO@A-TiO_2_/R-TiO_2_ nanocomposite photocatalyst via a facile sol–gel method. Several advanced characterization techniques were employed to systematically investigate the crystalline phases, morphology, elemental composition, and optical behavior of the nanocomposite. The photocatalytic activity evaluation revealed rapid degradation of the model dye pollutant Reactive Orange 16 under simulated solar light irradiation. The synergistic integration of narrow bandgap CuO with mixed phase TiO_2_ resulted in a Z-scheme mechanism that enabled effective spatial charge separation and inhibited recombination. This led to enhanced generation and longevity of reactive radical species, bolstering the photocatalytic activity. The two-pronged approach of incorporating visible light sensitization and retarding charge carrier recombination in the rationally designed nanocomposite proved highly effective. The present work successfully demonstrated the potential of carefully engineered multi-component nanocomposite photocatalysts for treatment of recalcitrant organic pollutants. This sets the stage for their practical application in large-scale water remediation under natural sunlight. Immediate future efforts should focus on exploring degradation of a diverse set of textile dyes, dye mixtures and real textile wastewater by the synthesized nanocomposite. Systematic studies evaluating the impacts of catalyst dose, pollutant concentration and water quality parameters would aid large-scale field deployment. From a materials development perspective, improving visible light absorption through co-catalyst loading and band structure modulation presents an exciting opportunity.

## Data Availability

The datasets used and analyzed during the current study are available from the corresponding author upon reasonable request.
